# Genome-Wide Identification and Characterization of the UMAMIT Gene Family in *Brassica napus*: Evolution, Expression, and Potential Association with Oil Content

**DOI:** 10.3390/ijms27198635

**Published:** 2026-09-26

**Authors:** Dan He, Tong Wang, Tongxiang Zhang, Xin He, Ping Qiu, Zhongsong Liu, Lunwen Qian

**Affiliations:** 1 College of Agronomy, Hunan Agricultural University, Changsha 410128, China; dd_yeah2021@163.com (D.H.); 15388025631@163.com (T.W.); 13617341408@163.com (T.Z.); hexinzhsh@126.com (X.H.); qiuping20230720@163.com (P.Q.); zsliu48@hunau.net (Z.L.); 2 Yuelushan Laboratory, Changsha 410128, China

**Keywords:** *Brassica napus*, amino acid transporter, UMAMIT, oil content, co-expression

## Abstract

The UMAMIT (Usually Multiple Acids Move In and Out Transporters) family comprises plant-specific amino acid transporters that play key roles in nitrogen uptake, transport, and storage. As a major oil crop in the Brassicaceae family, efficient amino acid transport in *Brassica napus* seeds is closely linked to seed yield and quality. Despite extensive studies in other species, the UMAMIT gene family has not been systematically characterized in *B. napus*. In this study, we identified 172 *BnaUMAMIT* genes, all of which harbor the EamA/WAT1-related domain and are predicted to localize to the plasma membrane. Both tandem and whole-genome/segmental duplications contributed to the expansion of this gene family. Phylogenetic analysis clustered BnaUMAMIT proteins into eight clades, revealing extensive collinearity with homologs in *Arabidopsis thaliana*, *Brassica rapa*, and *Brassica oleracea*. Gene structure analysis revealed conserved exon–intron organization within subgroups, whereas promoter analysis revealed diverse *cis*-acting elements associated with stress responses, hormone signaling, and developmental regulation. Transcriptome profiling revealed that 6 *BnaUMAMIT* genes were more highly expressed in low-oil-content seeds than in high-oil-content seeds at 25, 35, and 45 days after pollination in the two different environments, suggesting a possible association with seed oil content. Weighted gene co-expression network analysis (WGCNA) further demonstrated a significant correlative relationship between BnaUMAMIT transcripts and seed oil accumulation; meanwhile, co-expression network analysis revealed that *BnaUMAMITs* are correlated with the expression of lipid synthesis-related transcription factors, such as *BnaTT8*, *BnaSTK*, *BnaGL2*, *BnaLEC1*, and *BnaLEC2*, suggesting a potential regulatory association involved in seed oil accumulation. However, these results remain correlative and require further functional validation. Taken together, the results of this study provide a comprehensive understanding of the evolution, structure, and expression of the BnaUMAMIT gene family, laying a foundation for future functional studies and potential improvements in seed oil content in *B. napus*.

## 1. Introduction

Rapeseed (*Brassica napus* L., AACC, 2*n* = 38) is a significant oil crop worldwide. The high content of unsaturated fatty acids (FAs) in rapeseed oil has led to its widespread acceptance as both an edible vegetable oil for human consumption and a key feedstock for industrial biofuel production, which makes improving its seed oil content and nutritional quality a major breeding objective [1,2]. The seed oil content (SOC) in rapeseed is influenced by a wide spectrum of factors, including genetic background, climatic and edaphic conditions, and agronomic management practices. Oil accumulation in rapeseed is a sophisticated, tightly regulated metabolic process, whereby these factors exert their regulatory effects by modulating core biological processes, such as carbon–nitrogen metabolic homeostasis, the catalytic activities of key rate-limiting enzymes in lipid biosynthesis, the synthesis and phloem translocation of photosynthates, and embryonic development, which ultimately define the final level of seed oil accumulation [3,4,5].

It is a classic breeding topic in which protein content is often negatively correlated with oil content in seeds. Amino acids not only provide essential nitrogen for lipid biosynthesis but also supply metabolic intermediates, such as carnitine, that can be converted into fatty acid precursors through processes such as carbon chain elongation and dehydrogenation [6,7,8,9]. Many studies have shown that the synthesis of seed storage proteins and lipids occurs competitively, as these processes share the same substrates, energy supply, and storage space during seed development [10,11,12]. In *Arabidopsis thaliana*, it has been demonstrated that in *aap2* mutants, the source–sink translocation of amino acids is altered, resulting in decreased expression of storage proteins and elevated fatty acid levels [13]. *AtHISN1A* has also been shown to catalyze His biosynthesis, thereby regulating oil–protein balance. In *hisn1a* mutants, oil levels significantly decreased, whereas protein levels markedly increased [14]. In rapeseed and soybean, overexpression of the *Corynebacterium dapA* and *E. coli lysC* genes resulted in large increases in free lysine but a decrease in oil production [15].These findings suggest that although the regulatory mechanisms governing amino acid metabolism vary among plant species, this mode of regulation is prevalent, in which fatty acid biosynthesis is affected by the amino acid biosynthetic pathway.

Directional transport and accurate distribution of amino acids among cells, tissues, and organs directly influence key agronomic traits, including biomass accumulation, seed development, nutritional quality, and so forth [16,17,18,19,20]. Based on sequence characteristics and transport properties, plant amino acid transporters are generally categorized into three major families: the amino acid/auxin permease (AAAP) family, the amino acid/polyamine/choline transporter (APC) family, and the recently identified UMAMIT family [21,22]. Both the AAAP and APC families function mainly as unidirectional amino acid importers in plant cells [23,24]. In contrast, UMAMIT proteins are distinguished by their ability to mediate bidirectional amino acid efflux from intracellular compartments [25,26,27]. UMAMITs share homology with the *Escherichia coli* EamA-like amino acid transporter and belong to a distinct branch of the Nodulin-like (MtN21) gene family, characterized by the presence of a conserved EamA domain [28]. Functionally, UMAMITs are closely associated with bidirectional amino acid facilitators such as SIAR1 (Siliques Are Red1) and WAT1 (Walls-are-thin 1) [29,30].

The UMAMIT gene family has been most extensively characterized in the model plant *Arabidopsis thaliana*. In this species, 47 *UMAMIT* genes have been identified, three of which are considered pseudogenes because of the absence of transmembrane domains (TMDs). The remaining members are primarily localized to the tonoplast or plasma membrane and exhibit conserved amino acid export activity, facilitating the transport of metabolites along electrochemical gradients [29,31]. Several UMAMITs display tissue-specific expression and specialized functions. For instance, AtUMAMIT11 and AtUMAMIT14 are proposed to function cooperatively in phloem unloading, whereas AtUMAMIT28 and AtUMAMIT29 are critical for amino acid export from distinct symplastic domains during seed development [25]. AtUMAMIT24 and AtUMAMIT25 are also involved in amino acid transport during seed development, with AtUMAMIT24 contributing to temporary amino acid storage in the chalaza and AtUMAMIT25 mediating amino acid export from the endosperm [26]. Loss-of-function mutants of *AtUMAMIT44* exhibit reduced leaf area, decreased aboveground biomass, and significantly lower seed yield and protein content [32]. In addition, *AtUMAMIT29*, *30*, and *31* are specifically expressed in the funiculus and function as glucosinolate exporters, and disruption of these genes results in a dramatic reduction in seed glucosinolate accumulation [33]. Findings from existing studies reveal that the UMAMIT family constitutes a multifunctional transporter family that performs systemic functions in maintaining whole-plant carbon–nitrogen homeostasis. Spanning from source chloroplasts (UMAMIT44), phloem unloading in the roots (UMAMIT14/18), to the final sink organ seeds (UMAMIT24/25), this gene family plays distinct yet pivotal roles in the source–sink partitioning network of nitrogen. Their synergistic activities guarantee normal plant growth and development. During seed development, UMAMITs may modulate the efflux of amino acids from seeds or subcellular compartments, thereby shifting the metabolic flux between protein and oil synthesis. When the seeds are supplied with sufficient nitrogen, vigorous growth and storage protein biosynthesis occur. In contrast, under limited nitrogen availability with relatively abundant carbon resources, seed metabolism shifts toward oil accumulation, thereby indirectly affecting lipid accumulation.

The UMAMIT family facilitates bidirectional amino acid transport and serves as a core candidate for modulating seed carbon–nitrogen allocation. In addition, UMAMITs are key amino acid transporters that regulate amino acid accumulation and the transport of anti-nutritional factors such as glucosinolates during seed development, thereby affecting the nutritional value and processing quality of *Brassica napus*. The UMAMIT gene family has been identified and analyzed in a wide range of plant species. For example, 51, 213, 88, 48, 8, 16, and 13 *UMAMIT* genes have been identified in *Oryza sativa*, *Triticum aestivum*, *Medicago truncatula*, *Picea abies*, *Physcomitrella patens*, *Selaginella moellendorfii*, and *Pinus pinaster*, respectively [31,34]. However, a systematic genome-wide identification and analysis of the UMAMIT gene family in *B. napus* is still lacking, and the potential association between UMAMITs and rapeseed oil accumulation has rarely been investigated. Therefore, in this study, we systematically dissect the phylogeny and tissue expression profiles of *BnaUMAMITs* and their linkage to lipid deposition. This work bridges the knowledge gap in rapeseed, offers theoretical support for the manipulation of seed oil content through amino acid transport routes, and provides a foundation for future mechanistic studies.

## 2. Results

### 2.1. Identification and Chromosomal Location of the UMAMIT Gene Family in B. napus

The HMM profile of the EamA domain (PF00892) from the Pfam database, together with *Arabidopsis* UMAMIT (AtUMAMIT) amino acid sequences, was used as queries to identify homologous *BnaUMAMIT* genes in the *B. napus* genome. A total of 172 *BnaUMAMIT* genes were identified. All the encoded proteins possessed at least one EamA/WAT1-related domain, which is consistent with the fact that WAT1 (Walls Are Thin 1) proteins in plants typically harbor EamA domains (Table S1). Among these proteins, 165 contained the EamA domain, whereas 7 possessed only the WAT1-related domain.

Physicochemical analysis revealed that BnaUMAMIT proteins ranged from 108 to 738 amino acids, with 82% falling between 300 and 400 amino acids (Table S1). The molecular weights ranged from 32.82 to 44.21 kDa, and the predicted isoelectric points (pI) varied from 5.22 to 10.27, including 10 acidic (pI < 6.5), 156 alkaline (pI > 7.5), and 6 neutral (pI 6.5–7.5) proteins. All the proteins exhibited a positive grand average of hydropathicity. Subcellular localization predictions indicated that all BnaUMAMIT proteins are localized mainly to the plasma membrane, and some of them may also be localized in the vacuolar membrane, endoplasmic reticulum, chloroplast, and Golgi apparatus, which is consistent with findings in *Arabidopsis*, where UMAMITs exhibit diverse localization.

Among the 172 *BnaUMAMIT* genes, 129 were mapped to 19 chromosomes, whereas 43 were located on unanchored scaffolds (chrA01_random–chrCnn_random) (Figure 1). The genes exhibited a heterogeneous distribution across chromosomes, with 2 to 13 genes per chromosome. Nevertheless, these genes were relatively evenly distributed between the An subgenome (81 genes) and the Cn subgenome (91 genes). Among these genes, 45 underwent tandem duplication, whereas the remaining genes were generated through whole-genome duplication (WGD) or segmental duplication events, indicating that WGD/segmental duplication is the predominant mechanism driving BnaUMAMIT family expansion.

### 2.2. Phylogenetic and Collinearity Analysis of UMAMIT Proteins

In addition to *B. napus*, 78 and 87 putative UMAMIT proteins were identified in *B. rapa* and *B. oleracea*, respectively (Table S2). A phylogenetic tree for *A. thaliana*, *B. rapa*, *B. oleracea*, and *B. napus* was constructed by performing multiple sequence alignment of all UMAMIT protein sequences using MUSCLE and applying the neighbor-joining (NJ) method to investigate their evolutionary relationships (Figure 2). Phylogenetic analysis classified UMAMIT proteins across the Brassicaceae family into eight distinct clades. Group VII was the largest, comprising 42 BnaUMAMIT members, whereas Group VIII was the smallest, containing only six BnaUMAMIT members. Notably, each AtUMAMIT protein possesses at least one ortholog in *B. rapa*, *B. oleracea*, and *B. napus*, indicating strong evolutionary conservation of this transporter family across Brassica species.

To reconstruct the evolutionary history of the BnaUMAMIT gene family, we performed interspecific collinearity analysis across *B. napus*, *B. oleracea*, *B. rapa*, and *A. thaliana* (Figure 3). This analysis revealed extensive *UMAMIT* homology among these species. Notably, *B. napus* presented the greatest number of collinear gene pairs with *B. oleracea* (129 pairs), followed by *B. rapa* (119 pairs) and *A. thaliana* (60 pairs), which is consistent with the known allopolyploid origin of *B. napus* from *B. oleracea* and *B. rapa*. The An and Cn subgenomes of *B. napus* exhibit extensive collinearity with the diploid progenitor genomes of *B. rapa* (Ar genome) and *B. oleracea* (Co genome), respectively, and the majority of orthologous gene pairs retain conserved chromosomal positions. These findings indicate that whole-genome duplication was the primary mechanism underlying the rapid expansion of the BnaUMAMIT gene family; however, a subset of members likely originated from subsequent tandem or segmental duplications following ancestral WGD events.

### 2.3. Gene Structures and Conserved Motif Analysis of BnaUMAMIT Proteins

Analysis of gene structure is critical for elucidating gene function; thus, we comprehensively characterized the structure of the BnaUMAMIT gene family. On the basis of the architectural features of exon–intron organization, structural diversity among *BnaUMAMIT* genes appeared to reflect evolutionary divergence among homologs (Figure S1C). Within the same subgroup, most homologs exhibited comparable exon–intron structures, exon numbers, and gene lengths, with the most notable variations detected in intron and exon lengths. The conserved intron–exon structure within each subgroup provided robust support for both their close phylogenetic relationships and the classification of subfamilies; genes within the same clade tend to possess conserved functions and perform analogous amino acid transport activities. Nevertheless, considerable structural variations were observed among certain *BnaUMAMIT* genes within each subgroup, and those structural variations within the same phylogenetic subgroup may correlate with functional divergence. For example, in Group I, most genes contained six introns and seven exons; however, *BnaA07g14880D* contained 13 introns and 14 exons, which is likely due to segmental duplication events. This expanded structure may confer additional regulatory flexibility or enable alternative splicing. Conversely, *BnaC09g51760D* contained only two introns and three exons. Although *BnaC09g51760D* is substantially shorter than typical BnaUMAMIT proteins, it still contains a WAT1-related domain, suggesting that it represents a truncated but potentially functional isoform. This structural reduction, possibly caused by exon fusion, could alter its transmembrane domain topology and amino acid transport activity; however, functional assays are needed to test this hypothesis. These results suggesting that *BnaUMAMIT* genes may have undergone functional specialization through structural variation while retaining conserved core functions, and functional assays or transport activity assays are required to test this possibility in the future.

A total of 10 highly conserved motifs were identified in BnaUMAMIT proteins (Figure S1B). The canonical UMAMIT motif architecture follows the conserved linear order Motif3–Motif5–Motif2–Motif8–Motif4–Motif7–Motif9–Motif10–Motif1–Motif6, and both motif composition and copy number are highly consistent across UMAMIT proteins within the same phylogenetic clade (Table S1). In addition, some genes (e.g., *BnaA07g14880D* and *BnaA06g30670D*) contained duplicated motifs, which may increase protein stability or substrate binding affinity. In contrast, others (e.g., *BnaC09g51760D* and *BnaA04g10060D*) retained only approximately three conserved motifs, suggesting that it might have lost essential transport functions or evolved a different molecular role. These differences may be attributed to gene tandem duplication and gene fragment loss during the whole-genome duplication process.

### 2.4. Analysis of Cis-Acting Elements in the Promoter

To investigate the transcriptional regulatory mechanisms and infer potential biological functions of *BnaUMAMIT* genes, 2000-bp promoter sequences upstream of the translation start site (ATG) were extracted and systematically analyzed using the PlantCARE database. These elements were broadly grouped into three categories: abiotic and biotic stress responsiveness, phytohormone responsiveness, and plant growth and development (Table S3). In total, 4247 *cis*-acting elements were identified, including 1246 phytohormone-responsive elements, 776 abiotic/biotic stress-related elements, and 2225 elements related to plant growth and development. Among these *cis*-elements, light-responsive elements were the most abundant, accounting for 45.4% of the total. All *BnaUMAMIT* genes except *BnaA02g29170D* contained light-responsive elements, suggesting that the transcription of these genes may be potentially regulated by light signals. Phytohormone-responsive elements were the second most abundant category, with abscisic acid and methyl jasmonate response elements being predominant. In contrast, elements associated with seed-specific expression and palisade mesophyll cell differentiation were enriched exclusively in specific subgroups (Group VI and VII), suggesting that these genes may play specialized roles in tissue-specific processes, such as seed development, and reflect the temporal and spatial expression specificity of the BnaUMAMIT gene family.

### 2.5. Subcellular Localization of BnaUMAMIT Proteins

To validate the predicted subcellular localization, three BnaUMAMIT CDSs (without stop codons) were fused to GFP in pCam1304-SubC under the 35 S promoter and expressed in *N. benthamiana* leaves. Confocal laser microscopy revealed that free GFP (without a fusion gene) was distributed throughout the cells of *N. benthamiana*. In contrast, the GFP signal from BnaUMAMIT fusion proteins overlapped with that of the plasma membrane marker mCherry, indicating that these three BnaUMAMIT proteins are specifically localized to the plasma membrane (Figure 4). These results were consistent with the predicted subcellular localization, but considering the limited sample size, the subcellular localization of the remaining family members requires further experimental validation.

### 2.6. Expression Analysis of BnaUMAMIT Genes

Gene expression patterns and their regulatory networks collectively determine gene function. To investigate the roles of *BnaUMAMIT* genes during plant development, transcriptome data were analyzed using previously published datasets obtained from the BrassicaEDB (https://brassica.biodb.org/) database. These datasets were derived from the *Brassica napus* cultivar ‘Damor’ and encompassed multiple developmental stages as well as diverse organs and tissues. A gene expression heatmap revealed that members within the same subgroup generally exhibited similar expression patterns, whereas distinct differences were observed among different subgroups (Figure S2).

The expression levels of genes in Groups VIII and II were relatively low compared with those in the other subgroups, whereas the expression levels of most members of Group IV were consistently high across various tissues and developmental stages. Moreover, genes within the same subgroup often exhibit tissue-specific expression profiles. For instance, *BnaC08g46530D*, *BnaA10g08600D*, and *BnaA05g01920D* in Group VII were highly expressed in roots, stems, and mature siliques, whereas *BnaC04g05980D* and *BnaA05g34670D* from the same group were predominantly highly expressed in seeds during the podding and maturation stages. In Group VI, *BnaA09g48460D*, *BnaC02g46160D*, and *BnaA07g37180D* exhibited low or negligible expression in most tissues except for the seeds, whereas *BnaA09g51980D* was expressed mainly in the roots. Although Group IV genes were expressed in all the examined tissues, their expression levels were the highest in the stems. Notably, the expression of *BnaC01g11590D* and *BnaA04g11790D* was stable across all the tissues, indicating constitutive expression patterns. Collectively, these results demonstrate that *BnaUMAMIT* genes exhibit diverse tissue-specific expression patterns, whereas genes within the same subgroup display largely consistent expression profiles. This suggests the conservation of regulatory networks and functional mechanisms during evolution. Nevertheless, certain members appear to have undergone functional divergence during the course of evolution.

UMAMIT proteins are closely associated with amino acid transport during seed development. Therefore, the expression patterns of *BnaUMAMIT* genes at different seed development stages were further analyzed (Figure S3). All family members exhibited low expression during the seed germination stage, whereas most *BnaUMAMIT* genes were highly expressed during the podding stage. Elevated expression levels were particularly observed in the inner and outer integuments, as well as in the seeds and seed coats at late developmental stages. High expression levels were also detected in seeds and seed coats during maturation. Notably, the expression peaks of *BnaUMAMIT* genes coincided with the phase of nutrient accumulation during seed development, suggesting that these genes may play important roles in seed development and in nutrient synthesis and transport pathways.

### 2.7. Expression Patterns of BnaUMAMITs in Rapeseed Accessions with High and Low Oil Contents

To investigate the relationship between *BnaUMAMIT* gene expression and rapeseed oil content, RNA-seq data from two low-oil-content accessions (DY1 and DY2) and two high-oil-content accessions (GY1 and GY2) were analyzed. The seed oil contents of DY1 and DY2 were 34.5% and 37.5%, and their protein contents were 25.3% and 21.4%, respectively. While the seed oil contents of GY1 and GY2 were 50.1% and 51.4%, their protein contents were 20.0% and 19.9%, respectively. The samples were collected from Changsha (C) and Kunming (K) at 25, 35, and 45 days after pollination (DAP). The FPKM dataset was analyzed here to construct heatmaps examining the expression patterns of *BnaUMAMIT* genes (Figure S4). The expression profiles of 12 *BnaUMAMIT* genes revealed significant differences in expression between HOC (high oil content) and LOC (low oil content) accessions across different regions and developmental stages (Figure 5A). Among the 12 BnaUMAMIT genes, *BnaA05g34670D*, *BnaA06g18300D*, *BnaC04g47490D*, *BnaC03g55690D*, *BnaC04g33980D*, and *BnaC04g05980D* were more highly expressed in the LOC accessions than in the HOC accessions at 25, 35, and 45 DAP in the two different environments. To validate the RNA-seq results, qRT-PCR was performed on *BnaUMAMIT* genes across different seed developmental stages. The qRT-PCR results were consistent with the RNA–seq data (Figure 5B), confirming the reliability of the results of the transcriptomic analysis.

### 2.8. Co-Expression Analysis of BnaUMAMITs

To explore the regulatory relationships of *BnaUMAMIT* genes during seed development in rapeseed, weighted gene co-expression network analysis was performed using a filtered expression matrix comprising 20,822 genes derived from RNA-seq data. Based on expression similarity, these genes were clustered into 24 distinct modules, each represented by a unique color. The Pearson correlation coefficient (r > 0.5) and the corresponding *p* value (*p* < 0.05) were used to evaluate the relationship between module eigengenes and oil content (Figure S5). The results revealed that the pink module was significantly negatively correlated with high oil content, whereas the yellow and black modules were positively correlated with low oil content. Twenty-five *BnaUMAMIT* genes were distributed in four modules, namely, MEgray60, MEblue, MEsalmon, and MEpink, and all these modules were positively correlated with low oil content and negatively correlated with high oil content. Based on functional annotations, the BnaUMAMIT-associated co-expressed genes were further classified (Figure 6). Twenty-five *BnaUMAMIT* genes showed co-expression associations with 183 genes, including 22, 43, 34, 33, and 51 genes related to transcription factors, fatty acid metabolism, amino acid metabolism, and seed development, respectively.

## 3. Discussion

Amino acid transport is mediated by specialized transporter proteins in plants. As one of the amino acid transporter families, the UMAMIT family is capable of bidirectional transport and is considered a plant-specific transporter family whose core function is amino acid export [35,36,37,38,39]. In this study, we identified 78, 87, and 172 putative *UMAMIT* genes in *B. rapa*, *B. oleracea*, and *B. napus*, respectively. *B. napus* is an allotetraploid species (A_n_A_n_C_n_C_n_, 2*n* = 38) formed by hybridization between *B. rapa* (A genome) and *B. oleracea* (C genome), and its genome is characterized by extensive duplication events that often lead to functional diversification of homologous genes [40]. Consistent with this, the number of *BnaUMAMIT* genes exceeds the combined total found in its two diploid progenitors, indicating substantial expansion of this gene family during polyploidization. Chromosomal localization analysis further revealed the presence of tandem gene clusters on multiple chromosomes (chrA02, chrA03, chrA04, chrA07, chrA09, chrC02, chrC03, chrC04, chrC05, chrC06, and chrC07), suggesting that whole-genome duplication and segmental duplication were the primary forces driving BnaUMAMIT family expansion. Notably, compared with *A. thaliana*, *B. napus* has undergone multiple rounds of whole-genome triplication and duplication events, resulting in a genome approximately six times larger than that of *A. thaliana* [41]. Consequently, the number of *BnaUMAMIT* genes should theoretically be six times that in *A. thaliana*; however, the actual number is lower than expected. Furthermore, each *AtUMAMIT* gene has homologs in both the A and C subgenomes of *B. napus*, indicating that many *BnaUMAMIT* genes were lost during genome diploidization rather than during the WGT event. Phylogenetic analysis revealed that the BnaUMAMIT family could be divided into eight clades, which is consistent with previous classifications in Arabidopsis [31]. Synteny analysis among *A. thaliana*, *B. rapa*, *B. oleracea*, and *B. napus* further demonstrated that several *BnaUMAMIT* genes in the A and C subgenomes maintain collinear relationships with *UMAMIT* genes in their diploid progenitors and in *A. thaliana*. These results collectively indicate a high degree of evolutionary conservation within the *UMAMIT* gene family, thereby enabling functional inference of *BnaUMAMIT* genes based on well-characterized *AtUMAMIT* homologs.

Gene family expansion is often accompanied by the gain or loss of exons and introns [42]. Analyses of gene structures and conserved motifs revealed that most *BnaUMAMIT*s possess highly conserved motifs and share similar exon–intron architectures within the same subfamily. *BnaUMAMIT* genes contain primarily six introns and seven exons, and all the predicted BnaUMAMIT proteins harbor a typical EamA domain, further supporting the reliability of the phylogenetic grouping (Table S1). However, *BnaUMAMIT* genes with atypical exon–intron structures or altered motif compositions may have acquired distinct tissue-specific expression patterns or transportation functions. Notably, a small subset of BnaUMAMIT proteins was substantially shorter than the typical range. All these short proteins retained at least one EamA/WAT1-related domain, as verified by SMART and InterPro, indicating that they are unlikely to be completely non-functional pseudogenes or random annotation artifacts. However, their truncated lengths may result from incomplete gene annotation, partial gene fragments, or structural reduction through exon fusion, which could alter transmembrane domain topology and transport activity. The further evidence is required to determine whether they are functional truncated proteins or incomplete annotations. Physicochemical analysis revealed that BnaUMAMIT proteins are mainly alkaline proteins and exhibit a positive grand average of hydropathicity. These properties suggest that BnaUMAMIT proteins are likely basic, membrane-associated proteins that may anchor to membranes via their hydrophobic domains while interacting with intracellular anionic molecules or negatively charged membrane components [43]. The predicted subcellular localization, predominantly at the plasma membrane, some BnaUMAMITs were found located in vacuolar membrane, chloroplast, endoplasmic reticulum or Golgi apparatus, which is consistent with previous reports and further supports the involvement of BnaUMAMITs in amino acid transport processes [31].

*Cis*-acting regulatory elements located in promoter regions (upstream of the ATG start codon) play key roles in transcriptional regulation by responding to environmental and endogenous signals. Analysis of the *cis*-acting elements in the promoter regions of the BnUMAMIT gene family revealed that the promoter regions of all *BnaUMAMIT* genes contain multiple *cis*-acting elements, with substantial enrichment of elements linked to environmental responses (e.g., light, low temperature) and hormone signaling (e.g., MeJA, ABA) (Table S3). These findings suggest that the BnaUMAMIT gene family may not only be involved in basic amino acid transport but also likely integrate environmental and endogenous signals, thereby mediating *B. napus* adaptation to environmental stress and contributing to the regulation of growth and development. This interpretation is consistent with previous findings, which revealed that altered membrane amino acid transport can induce salicylic acid-mediated stress responses [44,45]. Moreover, the presence of seed- and endosperm-specific *cis*-elements suggests a possible role for UMAMITs in mediating amino acid transport to developing embryos and endosperm tissues [26].

*UMAMIT* genes exhibit distinct expression patterns across different tissues and development stages. Analysis of these spatial and temporal expression patterns provides a foundation for predicting their functions. Under normal growth conditions, most *BnaUMAMIT* genes display pronounced tissue-specific expression. For example, most Group IV genes are predominantly expressed in roots and stems, whereas genes such as *BnaC03g49570D*, *BnaA09g48460D*, and *BnaC06g12850D* are strongly expressed specifically in the seed. These patterns suggest that BnaUMAMITs, as amino acid transporters, may regulate growth and developmental processes in a tissue-dependent manner. To further explore their temporal expression, *BnaUMAMIT* gene expression was examined at different stages of seed development. Most genes are highly expressed in the inner/outer integuments and seed/seed coats during the podding stage and in the seed/seed coats during the late developmental and maturation stages. These expression patterns were largely consistent with those of their corresponding Arabidopsis homologs [25,46]. Notably, *BnaC02g37200D*, *BnaA09g02200D*, and *BnaC09g01610D* in Group I were expressed mainly during the early stages of seed development. These distinct temporal expression patterns indicate that certain *BnaUMAMIT* genes have undergone functional divergence during evolution and may play specialized roles at specific stages of seed development.

To investigate whether *BnaUMAMIT* expression is associated with seed oil content, we identified *BnaA05g34670D*, *BnaA06g18300D*, *BnaC04g47490D*, *BnaC03g55690D*, *BnaC04g33980D*, and *BnaC04g05980D*, whose expression levels were significantly greater in LOC rapeseed accessions than in HOC accessions, and the LOC accessions also had higher seed protein contents than the HOC accessions, consistent with the well-documented negative correlation between seed oil and protein content [47,48,49]. However, these relationships are correlative and may be influenced by genetic background; functional validation is required to determine whether *BnaUMAMIT*s directly affect carbon–nitrogen allocation and oil accumulation. Moreover, co-expression network analysis revealed that *BnaUMAMIT* genes exhibit co-expression associations with *BnaTT8*, *BnaSTK*, *BnaGL2*, *BnaLEC1*, and *BnaLEC2.* These transcription factors were found to be involved in the positive or negative regulation of the expression of genes responsible for the accumulation of oil [50,51,52,53]. These results suggesting that amino acid metabolism genes, oil accumulation-related transcription factors, and lipid metabolism genes have great potential to form a complex regulatory network to coordinately modulate seed oil content in rapeseed.

## 4. Materials and Methods

### 4.1. Identification of B. napus, B. rapa, and B. oleracea UMAMIT Genes

The protein sequences, coding sequences (CDS), and genomic sequences of 44 *AtUMAMIT* genes (excluding three pseudogenes) were retrieved from TAIR10 in TAIR (The Arabidopsis Information Resource, https://www.arabidopsis.org/ (accessed on 5 June 2023)) [54]. These sequences were used as queries to identify *UMAMIT* homologs in *B. rapa*, *B. oleracea*, and *B. napus* (Darmor-bzh) via BLASTP in the Brassicaceae Database (BRAD; http://brassicadb.cn/(accessed on 5 June 2023)) with thresholds of an E value < 1 × 10^−5^ and an identity > 50% [55]. Duplicated hits were removed, genes with an E value = 0 were retained, and a dataset from BLASTP was obtained.

To further validate the identified candidate proteins, the Hidden Markov Model (HMM) profile of the EamA domain (PF00892) was downloaded from InterPro (https://www.ebi.ac.uk/interpro/ (accessed on 8 June 2023)) [56] and used to scan the protein datasets of *B. napus*, *B. rapa*, and *B. oleracea* with HMMER v3.4 (http://hmmer.org (accessed on 8 June 2023)). Genes with E values < 1 × 10^−10^ were retained. The final *UMAMIT* gene set was determined by integrating the results from BLASTP and HMMER. Candidate genes were further verified for the presence of EamA/WAT1-related domains using the SMART database (http://smart.embl-heidelberg.de/ (accessed on 9 June 2023)) [57]. The molecular weight and isoelectric point of UMAMIT proteins were predicted using ExPASy (https://web.expasy.org/protparam/ (accessed on 18 October 2023)) and subcellular localization was predicted by ProtComp (http://linux1.softberry.com/ (accessed on 17 June 2023) site no longer accessible at present), WoLF PSORT (https://wolfpsort.hgc.jp/ (accessed on 11 June 2026)) and Plant-mPLoc (http://www.csbio.sjtu.edu.cn/bioinf/plant-multi/ (accessed on 11 June 2026)). By synthesizing all these results, we selected the two compartments with the highest scores for presentation.

### 4.2. Phylogenetic Analysis

Multiple sequence alignment of UMAMIT proteins from *A. thaliana*, *B. napus*, *B. rapa*, and *B. oleracea* was performed using MUSCLE in EMBL-EBI (https://www.ebi.ac.uk/jdispatcher/msa/muscle (accessed on 1 November 2023)) [58]. A neighbor-joining (NJ) phylogenetic tree was constructed in MEGA11 with 1000 bootstrap replicates, and the resulting tree was visualized and refined in R v4.3.1.

### 4.3. Gene Structure and Conserved Motif Analysis

Gene structures were analyzed using TBtools v2.3 [59] based on the GFF3 and CDS files of *A. thaliana*, *B. rapa*, *B. oleracea*, and *B. napus*. Conserved motifs were identified using the Multiple Expectation Maximization for Motif Elicitation (MEME) Suite (https://meme-suite.org/meme/tools/meme (accessed on 1 November 2023)), with the maximum number of motifs set to 10, motif occurrence per sequence set to 0 or 1, and other parameters at default settings. TBtools was used to visualize gene structures and motif distributions.

### 4.4. Chromosomal Localization and Collinearity Analysis

The chromosomal positions of the *UMAMIT* genes were determined using TBtools based on GTF/GFF annotation files for *A. thaliana*, *B. rapa*, *B. oleracea*, and *B. napus* downloaded from BRAD. Collinearity relationships among these genomes were analyzed with MCScanX (Multiple Collinearity Scan toolkit), and the results were visualized as a collinearity map using the Dual Synteny Plot function in TBtools.

### 4.5. Cis-Regulatory Element Analysis

The promoter sequences (2 kb upstream of the start codon) of *UMAMIT* genes were extracted using TBtools and analyzed for *cis*-acting elements using PlantCARE (https://bioinformatics.psb.ugent.be/webtools/plantcare/html/ (accessed on 1 November 2023)) [60]. The identified elements were filtered and visualized using the Simple BioSequence Viewer function in TBtools.

### 4.6. Subcellular Localization of BnaUMAMITs

The full-length coding sequences of the *BnaUMAMIT* genes used for subcellular localization were amplified from the *Brassica napus* cultivar ‘Damor-bzh’, which was provided by the College of Agronomy, Hunan Agricultural University (Changsha, China). *Nicotiana benthamiana* (wild-type) plants were also obtained from Hunan Agricultural University. The full-length CDS of three BnaUMAMIT proteins (excluding the stop codon) was cloned and inserted into the pCam1304-SubC vector fused to GFP under the CaMV 35 S promoter. All the primers used are listed in Table S4. The recombinant plasmids were subsequently introduced into *Agrobacterium tumefaciens* GV3101 via freeze–thaw transformation. Co-infiltration with organelle markers (PM-mCherry for the plasma membrane and NLS-mCherry for the nucleus) was performed in *Nicotiana benthamiana* leaves. Leaves were incubated for 24 h in the dark, followed by 24 h under low light, after which GFP fluorescence was observed using a confocal laser microscope (Leica SP8 SR, Wetzlar, Germany). Leaves transformed with the 35S:GFP vector alone served as controls.

### 4.7. Tissue-Specific Expression Analysis of BnUMAMIT Family Genes

The expression profiles of 106 *BnaUMAMIT* genes across various tissues and developmental stages were obtained from BrassicaEDB (https://brassica.biodb.org/ (accessed on 9 December 2023)) [61]. Gene IDs were entered into the search box, and the eFP database was selected to retrieve gene expression levels across the full growth cycle and different tissues of *Brassica napus*. The transcriptome data covered 6 tissues (root, leaf, seed, stem, flower, and silique) and 7 developmental stages (germination, seedling, bolting, initial flowering, full-bloom, podding, and maturation), as well as post-pollination stages of seed development. The expression values were normalized to a log2 scale and visualized using TBtools.

The FPKM (fragments per kilobase of transcript per million fragments mapped) values of *BnaUMAMIT* genes from two low-oil-content (renamed DY1 and DY2) and two high-oil-content (renamed GY1 and GY2) accessions were obtained from RNA-Seq data in our previous study [62]. The seed oil contents of DY1(Xiangyou 774) and DY2(Xiangyou 15) were 34.5% and 37.5%, and their protein contents were 25.3% and 21.4%, respectively. While the seed oil contents of GY1(ZY036) and GY2(ZY511) were 50.1% and 51.4%, their protein contents were 20.0% and 19.9%, respectively, as measured by near-infrared reflectance spectroscopy. These four accessions were grown in Changsha (C) and Kunming (K), and seed samples were collected at 25, 35, and 45 days after pollination (DAP), with three independent biological replicates collected from separate plants for each accession, environment, and time point. Genes with a |log_2_(fold change)| ≥ 1 and an FDR adjusted *p* value < 0.05 were considered to be differentially expressed. Heatmaps of *BnaUMAMIT* gene expression profiles were generated using TBtools.

Six *BnaUMAMIT* genes were selected for validation by qRT-PCR. First-strand cDNA was synthesized using PrimeScript RT Master Mix (TaKaRa Biotech, Dalian, China) according to the manufacturer’s instructions. qRT-PCR reactions were performed on a CFX96 Touch Real-Time PCR detection system using SYBR Green Supermix (Bio-Rad Laboratories, Hercules, CA, USA). The PCR reactions, cycling protocol and melting curves were performed according to the manufacturer’s instructions. Three biological replicates and three measurements for each replicate were performed under identical conditions. *BnaActin7* was used to normalize the expression levels of the target genes, which were analyzed using the relative quantification method 2^−ΔΔCt^. All primers used in this study are listed in Table S4.

### 4.8. Co-Expression Network Analysis

Weighted gene co-expression network analysis (WGCNA) was performed to analyze the relationships between BnaUMAMITs and other proteins based on gene expression profiles. The gene co-expression network was constructed using the WGCNA-ShinyApp function in TBtools. Genes with an average FPKM < 0.1 were filtered out to ensure a high-quality network. To construct a co-expression network, we first determined the appropriate soft-thresholding power by evaluating the scale-free topology fit index across a range of candidate powers (1–30). A soft-thresholding power of 10 was selected, which yielded a scale-free topology fit index (R^2^) of 0.78 and a slope of −1.41, indicating a reasonably good approximation to scale-free topology consistent with biological expectations (R^2^ > 0.80). Higher powers (e.g., 12) yielded a lower R^2^ or led to over-aggressive pruning of weak correlations. We therefore selected a power of 10 as a balance between maintaining a biologically meaningful network and preserving sufficient connectivity. This power was subsequently used to calculate the adjacency matrix and derive topological overlap for module detection. Genes with similar expression patterns were grouped into distinct modules, and Pearson correlation analysis was used to evaluate correlations between module-specific genes and oil content. The co-expression network was visualized using Cytoscape version 3.10 [63].

## 5. Conclusions

In this study, we identified 172 *BnaUMAMIT* genes in *B. napus* and classified them into eight phylogenetic subgroups. Promoter regions harbor diverse *cis*-acting elements, indicating that *BnaUMAMIT* genes may integrate environmental and hormonal signals to modulate growth and development. Expression profiling revealed that *BnaA05g34670D*, *BnaA06g18300D*, *BnaC04g47490D*, *BnaC03g55690D*, *BnaC04g33980D*, and *BnaC04g05980D* were consistently highly expressed in LOC accessions across different environments and seed developmental stages. Co-expression network analysis further demonstrated that these genes are correlated with lipid metabolism, seed development, and amino acid transport pathways and are associated with key transcription factors that govern oil accumulation. Notably, the potential relationship between *BnaUMAMIT* genes and seed oil content needs to be further verified. Subsequent genetic functional experiments, such as gene knockout, heterologous overexpression, elemental analysis of carbon and nitrogen, and seed-specific expression analyses, are essential to clarify the exact function and regulatory mechanism of BnaUMAMITs in modulating rapeseed seed oil content. This study provides a comprehensive foundation for future functional characterization and potential genetic improvement of the seed oil content in rapeseed.

## Figures and Tables

**Figure 1:**
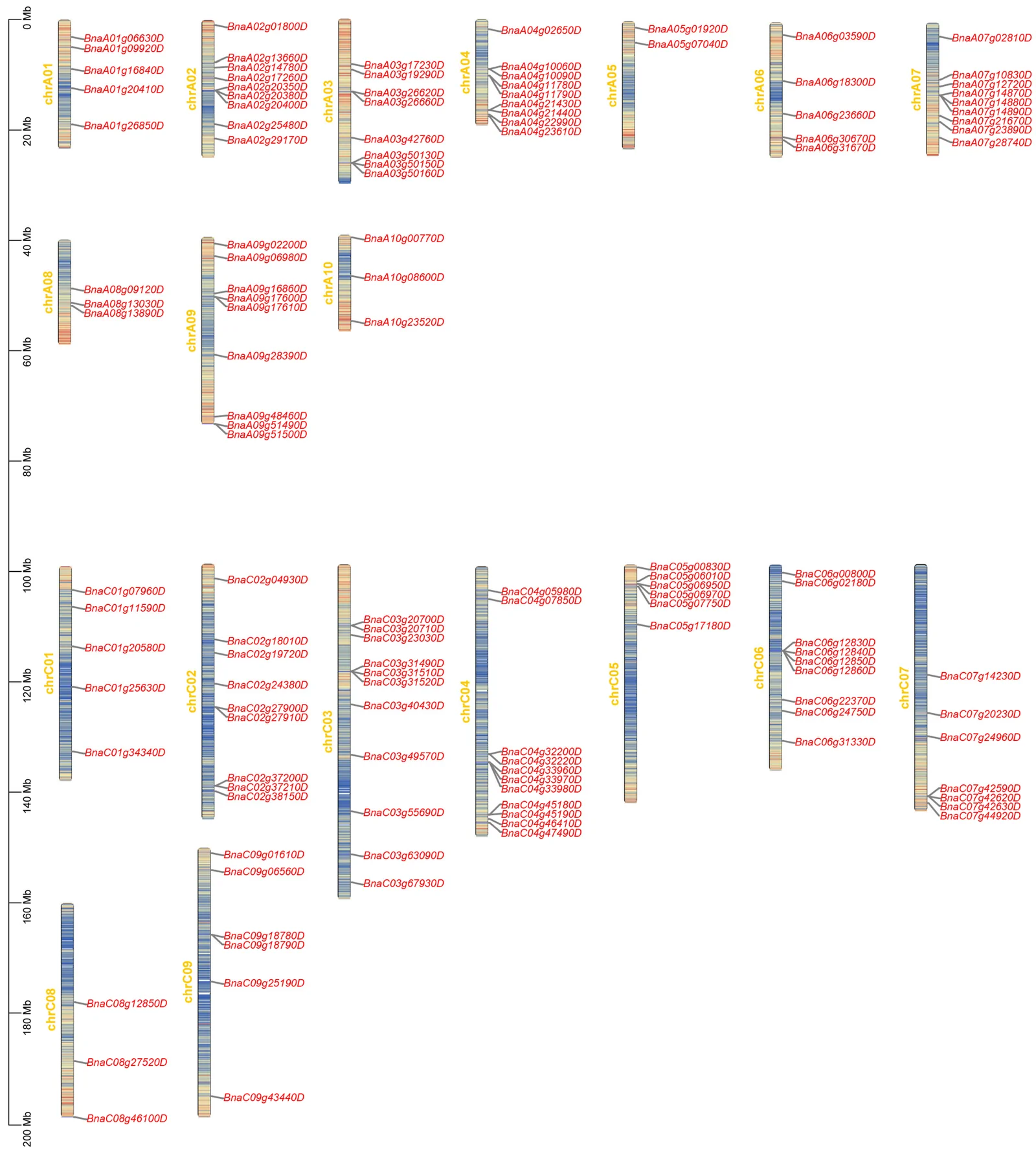
Distribution of *UMAMIT* genes on *B. napus* chromosomes. chrA01–10: An-subgenome chromosomes; chrC01–09: Cn-subgenome chromosomes. The color on the chromosome indicates the gene density of all the genes. The redder color indicates the higher gene density, while the bluer color indicates lower gene density.

**Figure 2:**
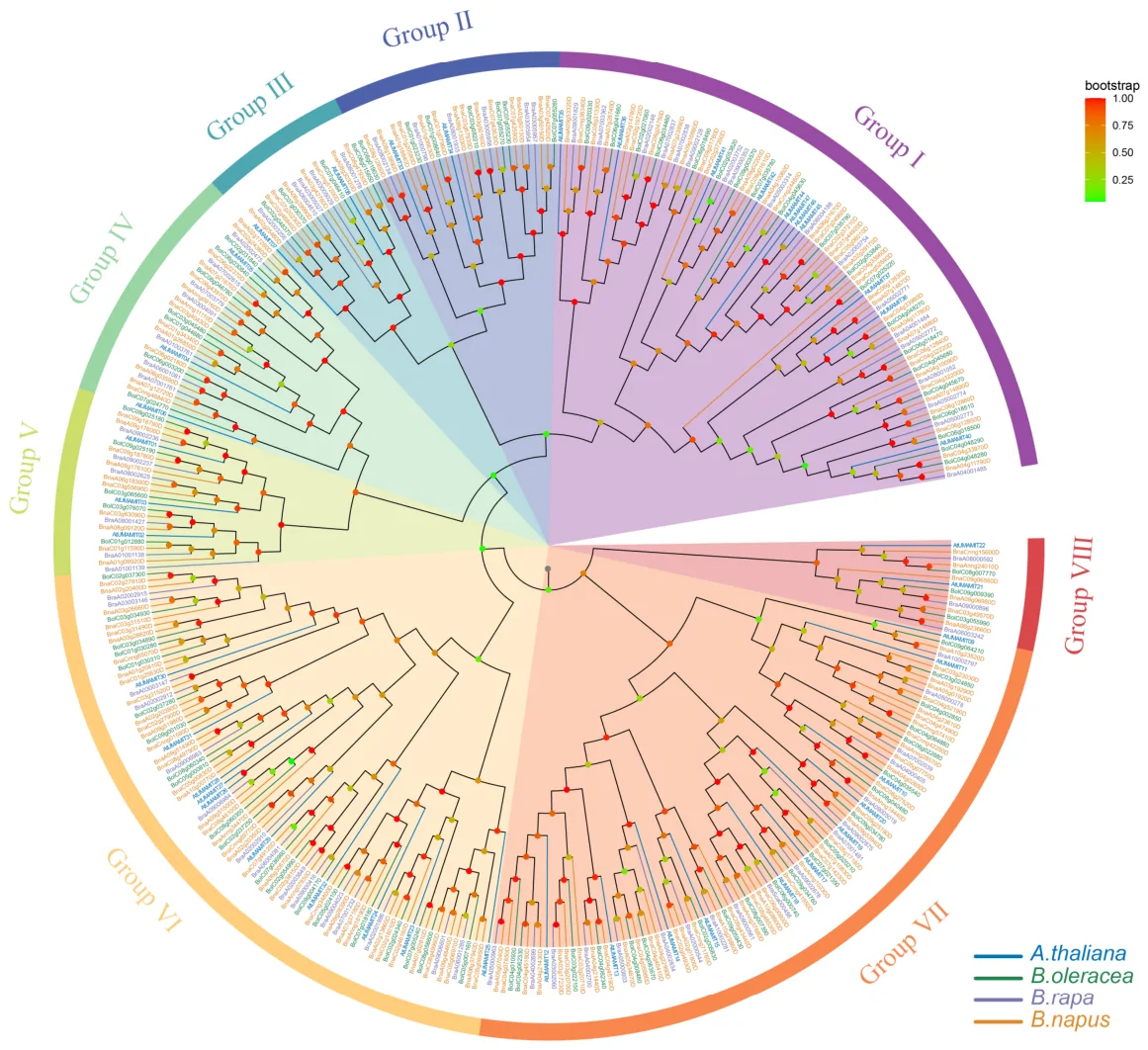
Phylogenetic tree of UMAMIT proteins from *A. thaliana*, *B. rapa*, *B. oleracea*, and *B. napus*. A neighbor-joining phylogenetic tree was generated by MEGA11 with full-length UMAMIT protein sequences (1000 bootstrap replicates). Colored dots at branch nodes represent bootstrap values, the color gradient shifts from green to red, corresponding to an increase in bootstrap. The UMAMITs from *A. thaliana*, *B. rapa*, *B. oleracea*, and *B. napus* are indicated by blue, violet, green, and orange labels, respectively. The eight resulting groups (Group I–Group VIII) are highlighted by different colors.

**Figure 3:**
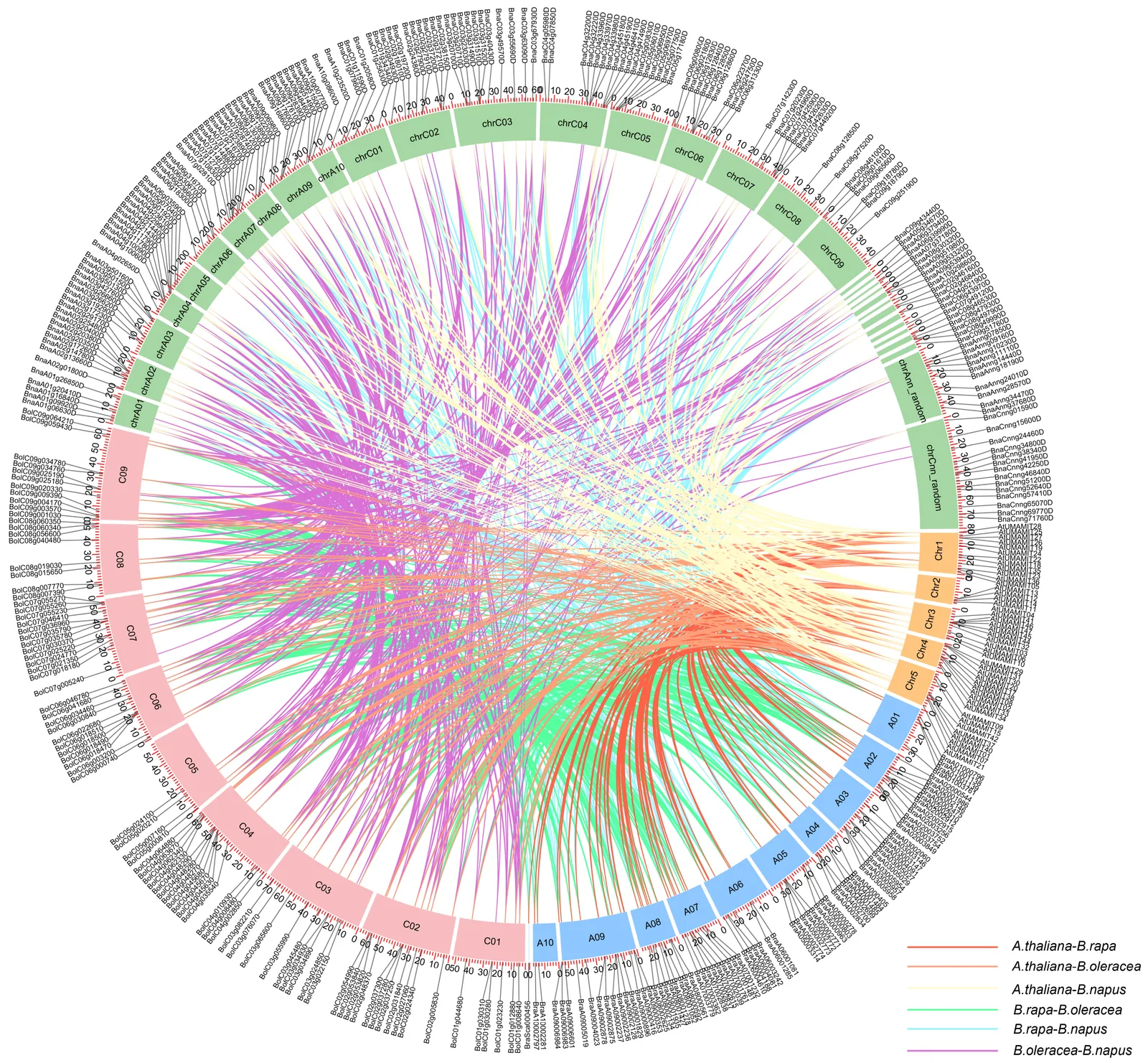
Syntenic relationships among *UMAMIT*s of *A. thaliana*, *B. rapa*, *B. oleracea*, and *B. napus*. The chromosomes of *A. thaliana*, *B. rapa*, *B. oleracea*, and *B. napus* are shown in orange, blue, pink, and green, respectively. The orthologous and paralogous *UMAMIT* genes were mapped onto the chromosomes/scaffolds and linked to each other. The syntenic *UMAMIT* gene pairs from *A. thaliana* and *B. rapa*, *A. thaliana* and *B. oleracea*; *A. thaliana* and *B. napus*; *B. rapa* and *B. oleracea*; and *B. rapa* and *B. napus*, *B. oleracea* and *B. napus* are linked by red, orange, yellow, green, blue, and purple lines, respectively.

**Figure 4:**
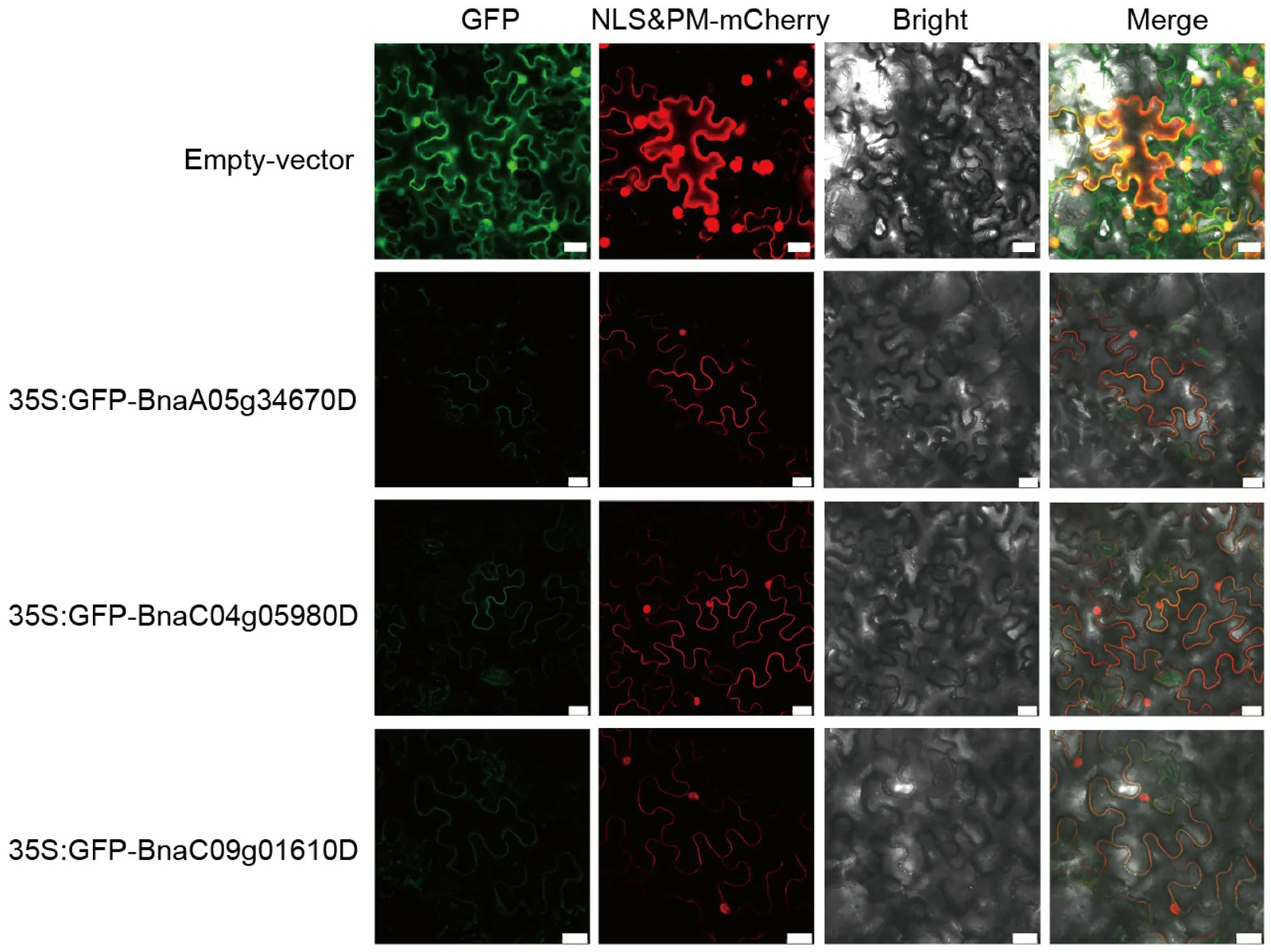
Subcellular location of the BnaUMAMIT fusion protein. NLS protein fused with mCherry was used as a nuclear marker, and PM protein fused with mCherry was used as a plasma membrane marker. Leaves transformed with the 35S:GFP vector (empty vector) alone were used as a control. Scale bar = 25 μm.

**Figure 5:**
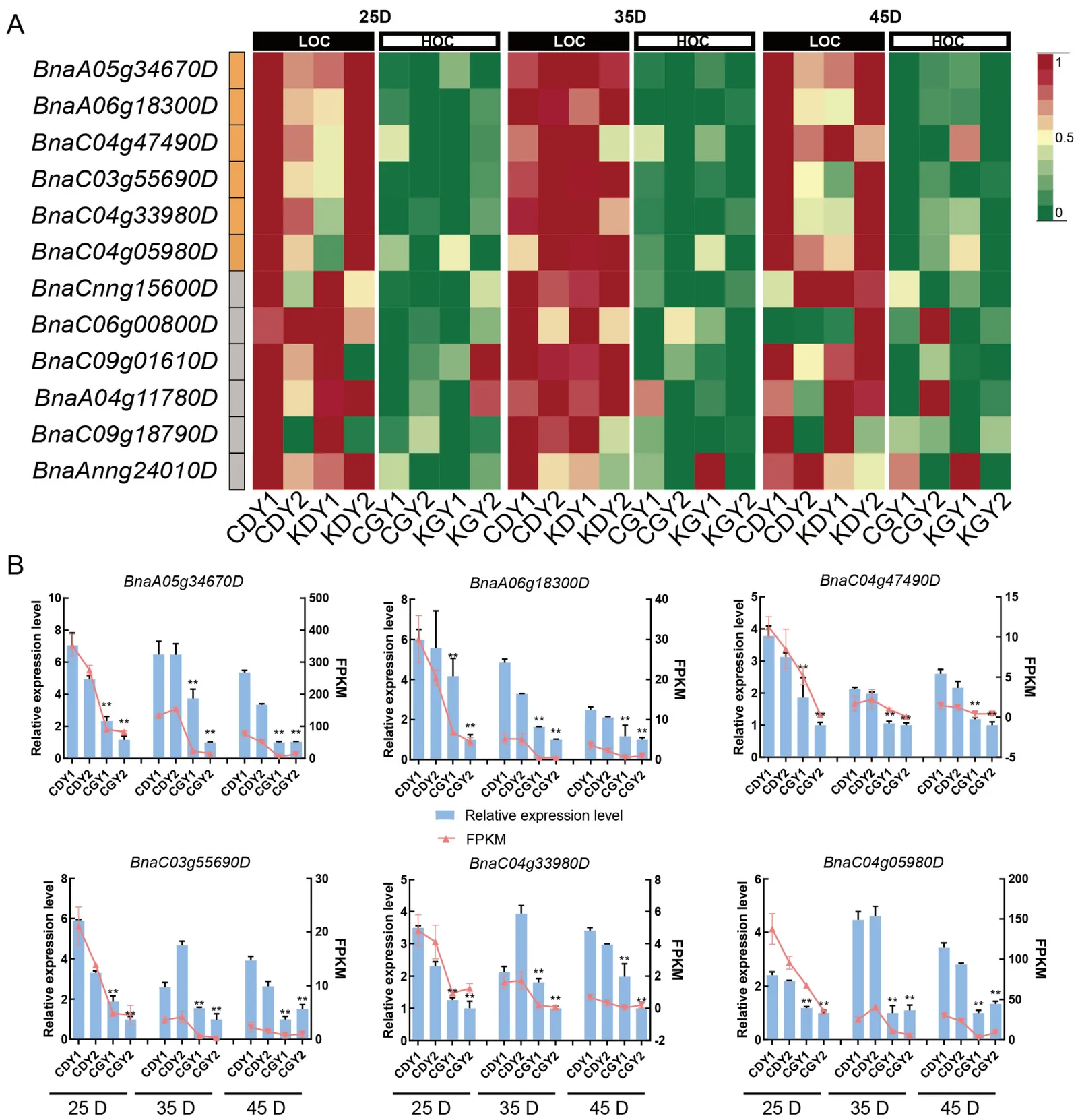
Expression patterns of *BnaUMAMIT* genes in high- and low-oil-content *B. napus* seeds from Changsha (C) and Kunming (K) at 25, 35, and 45 days. (**A**) The heatmap is generated by comparing the relative results of different genes in different periods. Red, green, and yellow indicate high, low, and medium expression levels, respectively. The orange squares next to gene IDs represent genes that are more highly expressed in the LOC accessions than in the HOC accessions. LOC: low oil content, HOC: high oil content. CDY1/2 and KDY1/2 represent low-oil-content seeds; CGY1/2 and KGY1/2 represent high-oil-content seeds. (**B**) qRT-PCR analysis of relative expression levels. The relative qRT-PCR expression levels are shown on the left y-axis, and the results are presented as the mean ± SD of three technical replicates. The FPKM values are shown on the right y-axis, and the results are presented as the mean ± SD of three biological replicates. Asterisks indicate significant differences (Student’s *t* test; ** indicates *p* < 0.01).

**Figure 6:**
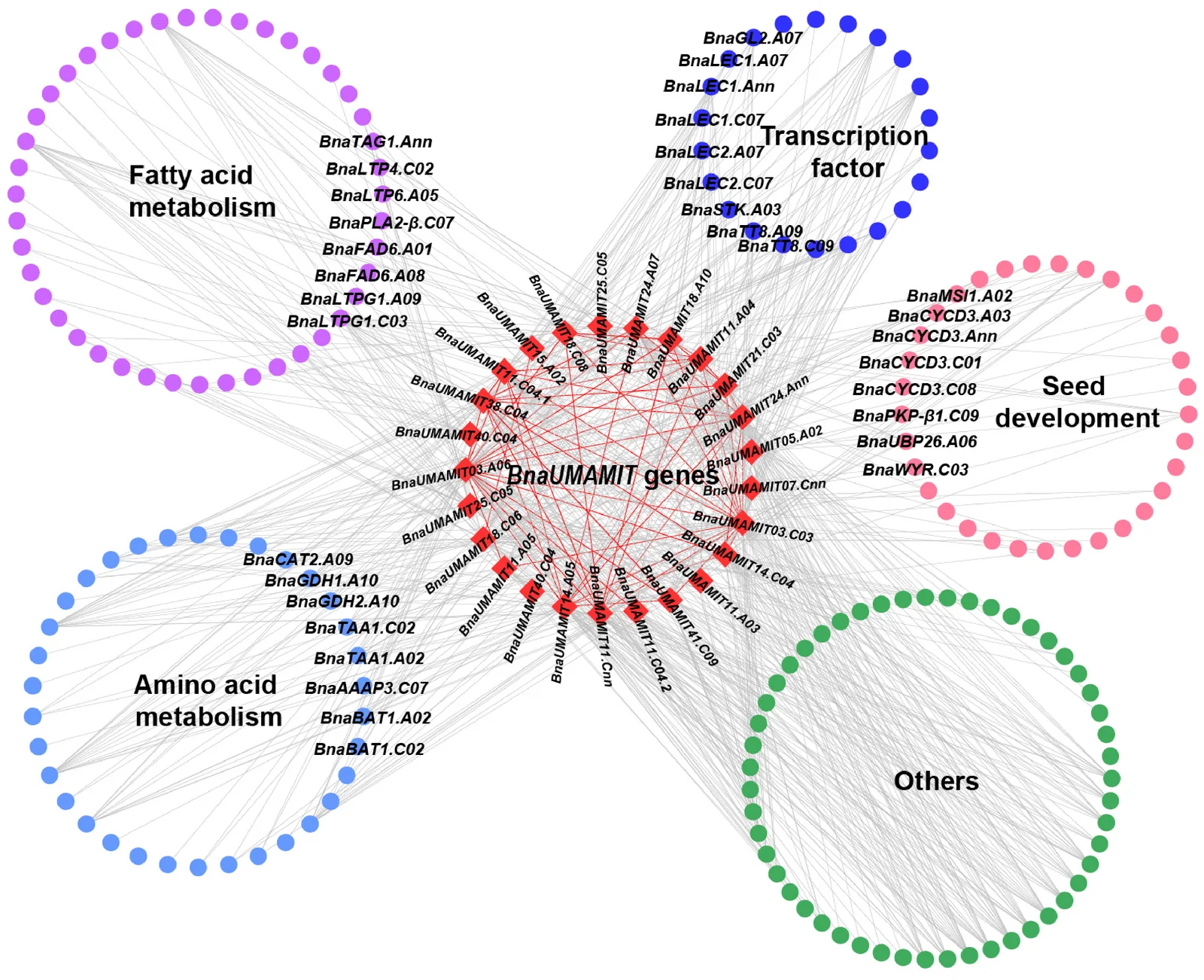
Co-expression network analysis of *BnaUMAMIT* genes. The red rhombuses represent *BnaUMAMIT* genes, and different pathway-related genes are colored differently. Only the colors of the modules where the UMAMIT genes are located are shown in the figure, and they are gray, blue, salmon, and pink.

## Data Availability

The original contributions presented in this study are included in the article. Further inquiries can be directed to the corresponding author.

## Supplementary Materials

The following supporting information can be downloaded at: https://www.mdpi.com/article/10.3390/ijms27198635/s1.

## References

[1] Friedt W., Tu J., Fu T., Liu S., Snowdon R., Chalhoub B. (2018). Academic and economic importance of *Brassica napus* rapeseed. The Brassica napus Genome.

[2] Rathnakumar A.L., Sujatha M., Gosal S.S., Wani S.H. (2022). Breeding major oilseed crops: Prospects and future research needs. Accelerated Plant Breeding, Volume 4: Oil Crops.

[3] Tang S., Zhao H., Lu S., Yu L., Zhang G., Zhang Y., Yang Q.Y., Zhou Y., Wang X., Ma W. (2021). Genome- and transcriptome-wide association studies provide insights into the genetic basis of natural variation of seed oil content in *Brassica napus*. Mol. Plant.

[4] Han X., Wu X., Zhang Y., Tang Q., Zeng L., Liu Y., Xiang Y., Hou K., Fang S., Lei W. (2025). Genetic and transcriptome analyses of the effect of genotype-by-environment interactions on *Brassica napus* seed oil content. Plant Cell.

[5] Shen W., Yu L., Qu Q., Han X., Ma W., Zu F., Guo L., Tang S. (2025). Genetic optimization of the source, sink and flow for increasing seed oil content in rapeseed. J. Integr. Plant Biol..

[6] Bourdin B., Adenier H., Perrin Y. (2007). Carnitine is associated with fatty acid metabolism in plants. Plant Physiol. Biochem..

[7] Zhang W., Zhang Z., Yan S. (2015). Effects of various amino acids as organic nitrogen sources on the growth and biochemical composition of *Chlorella pyrenoidosa*. Bioresour. Technol..

[8] Jacques F., Rippa S., Perrin Y. (2018). Physiology of L-carnitine in plants in light of the knowledge in animals and microorganisms. Plant Sci..

[9] Cai Y., Chen H., Tang X., Zhao J., Zhang H., Chen Y.Q., Chen W. (2022). The relationship between amino acid and lipid metabolism in oleaginous eukaryotic microorganism. Appl. Microbiol. Biotechnol..

[10] Si P., Mailer R.J., Galwey N., Turner D.W. (2003). Influence of genotype and environment on oil and protein concentrations of canola (*Brassica napus* L.) grown across southern Australia. Aust. J. Agric. Res..

[11] Borisjuk L., Neuberger T., Schwender J., Heinzel N., Sunderhaus S., Fuchs J., Hay J.O., Tschiersch H., Braun H.P., Denolf P. (2013). Seed architecture shapes embryo metabolism in oilseed rape. Plant Cell.

[12] Chao H., Wang H., Wang X., Guo L., Gu J., Zhao W., Li B., Chen D., Raboanatahiry N., Li M. (2017). Genetic dissection of seed oil and protein content and identification of networks associated with oil content in *Brassica napus*. Sci. Rep..

[13] Zhang L., Tan Q., Lee R., Trethewy A., Lee Y.H., Tegeder M. (2010). Altered xylem-phloem transfer of amino acids affects metabolism and leads to increased seed yield and oil content in *Arabidopsis*. Plant Cell.

[14] Ma H., Wang S. (2016). Histidine regulates seed oil deposition through abscisic acid biosynthesis and β-oxidation1. Plant Physiol..

[15] Falco S.C., Guida T., Locke M., Mauvais J., Sanders C., Ward R.T., Webber P. (1995). Transgenic canola and soybean seeds with increased lysine. Bio/Technology.

[16] Ortiz-Lopez A., Chang H.C., Bush D.R. (2000). Amino acid transporters in plants. Biochim. Biophys. Acta.

[17] Schroeder J.I., Delhaize E., Frommer W.B., Guerinot M.L., Harrison M.J., Herrera-Estrella L., Horie T., Kochian L.V., Munns R., Nishizawa N.K. (2013). Using membrane transporters to improve crops for sustainable food production. Nature.

[18] Dinkeloo K., Boyd S., Pilot G. (2018). Update on amino acid transporter functions and on possible amino acid sensing mechanisms in plants. Semin. Cell Dev. Biol..

[19] Guo N., Zhang S., Gu M., Xu G. (2021). Function, transport, and regulation of amino acids: What is missing in rice?. Crop J..

[20] Xu D., Sanden N.C.H., Hansen L.L., Belew Z.M., Madsen S.R., Meyer L., Jørgensen M.E., Hunziker P., Veres D., Crocoll C. (2023). Export of defensive glucosinolates is key for their accumulation in seeds. Nature.

[21] Yang G., Wei Q., Huang H., Xia J. (2020). Amino acid transporters in plant cells: A brief review. Plants.

[22] Yao X., Nie J., Bai R., Sui X. (2020). Amino acid transporters in plants: Identification and function. Plants.

[23] Svennerstam H., Ganeteg U., Näsholm T. (2008). Root uptake of cationic amino acids by Arabidopsis depends on functional expression of amino acid permease 5. New Phytol..

[24] Svennerstam H., Jämtgård S., Ahmad I., Huss-Danell K., Näsholm T., Ganeteg U. (2011). Transporters in Arabidopsis roots mediating uptake of amino acids at naturally occurring concentrations. New Phytol..

[25] Müller B., Fastner A., Karmann J., Mansch V., Hoffmann T., Schwab W., Suter-Grotemeyer M., Rentsch D., Truernit E., Ladwig F. (2015). Amino acid export in developing Arabidopsis seeds depends on UmamiT facilitators. Curr. Biol..

[26] Besnard J., Zhao C., Avice J.C., Vitha S., Hyodo A., Pilot G., Okumoto S. (2018). Arabidopsis UMAMIT24 and UMAMIT25 are amino acid exporters involved in seed loading. J. Exp. Bot..

[27] Cao C., Qiu X., Yang Z., Jin Y. (2025). New insights into the evolution and function of the UMAMIT (usually multiple acids move in and out transporter) gene family. J. Plant Res..

[28] Denancé N., Szurek B., Noël L.D. (2014). Emerging functions of nodulin-like proteins in non-nodulating plant species. Plant Cell Physiol..

[29] Ladwig F., Stahl M., Ludewig U., Hirner A.A., Hammes U.Z., Stadler R., Harter K., Koch W. (2012). SIARed1 from Arabidopsis acts as a bidirectional amino acid transporter that is crucial for the amino acid homeostasis of siliques. Plant Physiol..

[30] Ranocha P., Dima O., Nagy R., Felten J., Corratgé-Faillie C., Novák O., Morreel K., Lacombe B., Martinez Y., Pfrunder S. (2013). Arabidopsis WAT1 is a vacuolar auxin transport facilitator required for auxin homoeostasis. Nat. Commun..

[31] Zhao C., Pratelli R., Yu S., Shelley B., Collakova E., Pilot G. (2021). Detailed characterization of the UMAMIT proteins provides insight into their evolution, amino acid transport properties, and role in the plant. J. Exp. Bot..

[32] The S.V., Santiago J.P., Pappenberger C., Hammes U.Z., Tegeder M. (2024). UMAMIT44 is a key player in glutamate export from Arabidopsis chloroplasts. Plant Cell.

[33] Meyer L., Crocoll C., Halkier B.A., Mirza O.A., Xu D. (2023). Identification of key amino acid residues in AtUMAMIT29 for transport of glucosinolates. Front. Plant Sci..

[34] Fang Z.T., Kapoor R., Datta A., Okumoto S. (2022). Tissue specific expression of UMAMIT amino acid transporters in wheat. Sci. Rep..

[35] Lee Y.H., Foster J., Chen J., Voll L.M., Weber A.P.M., Tegeder M. (2007). AAP1 transports uncharged amino acids into roots of Arabidopsis. Plant J..

[36] Sanders A., Collier R., Trethewy A., Gould G., Sieker R., Tegeder M. (2009). AAP1 regulates import of amino acids into developing Arabidopsis embryos. Plant J..

[37] Denancé N., Ranocha P., Martinez Y., Sundberg B., Goffner D. (2010). Light-regulated compensation of *wat1* (*walls are thin1*) growth and secondary cell wall phenotypes is auxin-independent. Plant Signal. Behav..

[38] Ranocha P., Denancé N., Vanholme R., Freydier A., Martinez Y., Hoffmann L., Köhler L., Pouzet C., Renou J.P., Sundberg B. (2010). Walls are thin 1 (WAT1), an Arabidopsis homolog of Medicago truncatula NODULIN21, is a tonoplast-localized protein required for secondary wall formation in fibers. Plant J..

[39] Yang H., Krebs M., Stierhof Y.D., Ludewig U. (2014). Characterization of the putative amino acid transporter genes *AtCAT2*, *3* & *4*: The tonoplast localized AtCAT2 regulates soluble leaf amino acids. J. Plant Physiol..

[40] Chalhoub B., Denoeud F., Liu S., Parkin I.A.P., Tang H., Wang X., Chiquet J., Belcram H., Tong C., Samans B. (2014). Early allopolyploid evolution in the post-Neolithic *Brassica napus* oilseed genome. Science.

[41] Parkin I.A.P., Gulden S.M., Sharpe A.G., Lukens L., Trick M., Osborn T.C., Lydiate D.J. (2005). Segmental structure of the *Brassica napus* genome based on comparative analysis with *Arabidopsis thaliana*. Genetics.

[42] Xu G., Guo C., Shan H., Kong H. (2012). Divergence of duplicate genes in exon–intron structure. Proc. Natl. Acad. Sci. USA.

[43] Ilgü H., Jeckelmann J.M., Kalbermatter D., Ucurum Z., Lemmin T., Fotiadis D. (2021). High-resolution structure of the amino acid transporter AdiC reveals insights into the role of water molecules and networks in oligomerization and substrate binding. BMC Biol..

[44] Besnard J., Sonawala U., Maharjan B., Collakova E., Finlayson S.A., Pilot G., McDowell J., Okumoto S. (2021). Increased expression of UMAMIT amino acid transporters results in activation of salicylic acid-dependent stress response. Front. Plant Sci..

[45] Wang R., Zhong Y., Liu X., Zhao C., Zhao J., Li M., Ul Hassan M., Yang B., Li D., Liu R. (2021). Cis-regulation of the amino acid transporter genes *ZmAAP2* and *ZmLHT1* by ZmPHR1 transcription factors in maize ear under phosphate limitation. J. Exp. Bot..

[46] Besnard J., Pratelli R., Zhao C., Sonawala U., Collakova E., Pilot G., Okumoto S. (2016). UMAMIT14 is an amino acid exporter involved in phloem unloading in Arabidopsis roots. J. Exp. Bot..

[47] Katepa-Mupondwa F., Raney J.P., Rakow G. (2005). Recurrent selection for increased protein content in yellow mustard (*Sinapis alba* L.). Plant Breed..

[48] Weselake R.J., Taylor D.C., Rahman M.H., Shah S., Laroche A., McVetty P.B.E., Harwood J.L. (2009). Increasing the flow of carbon into seed oil. Biotechnol. Adv..

[49] Zhang Z., Dunwell J.M., Zhang Y.M. (2018). An integrated omics analysis reveals molecular mechanisms that are associated with differences in seed oil content between *Glycine max* and *Brassica napus*. BMC Plant Biol..

[50] Baud S., Mendoza M.S., To A., Harscoët E., Lepiniec L., Dubreucq B. (2007). WRINKLED1 specifies the regulatory action of LEAFY COTYLEDON2 towards fatty acid metabolism during seed maturation in *Arabidopsis*. Plant J..

[51] Elahi N., Duncan R.W., Stasolla C. (2016). Modification of oil and glucosinolate content in canola seeds with altered expression of *Brassica napus LEAFY COTYLEDON1*. Plant Physiol. Biochem..

[52] Qian L., Yang L., Liu X., Wang T., Kang L., Chen H., Lu Y., Zhang Y., Yang S., You L. (2025). Natural variations in *TT8* and its neighboring *STK* confer yellow seed with elevated oil content in *Brassica juncea*. Proc. Natl. Acad. Sci. USA.

[53] Bai Z., Sun H., Shi W., Zhou C., Hu M., Xie M., Gao F., Tong C., Liu Y., Chai G. (2026). Joint analysis of GWAS and TILLING reveals the negative role of GLABRA2 in controlling seed oil content in *Brassica napus*. Theor. Appl. Genet..

[54] Reiser L., Bakker E., Subramaniam S., Chen X., Sawant S., Khosa K., Prithvi T., Berardini T.Z. (2024). TAIR: The Arabidopsis information resource in 2024. Genetics.

[55] Chen H., Wang T., He X., Cai X., Lin R., Liang J., Wu J., King G., Wang X. (2022). BRAD V3.0: An upgraded Brassicaceae database. Nucleic Acids Res..

[56] Blum M., Andreeva A., Florentino L.C., Chuguransky S.R., Grego T., Hobbs E., Pinto B.L., Orr A., Paysan-Lafosse T., Ponamareva I. (2025). InterPro: The protein sequence classification resource in 2025. Nucleic Acids Res..

[57] Letunic I., Doerks T., Bork P. (2015). SMART: Recent updates, new developments and status in 2015. Nucleic Acids Res..

[58] Madeira F., Madhusoodanan N., Lee J., Eusebi A., Niewielska A., Tivey A.R.N., Lopez R., Butcher S. (2024). The EMBL-EBI job dispatcher sequence analysis tools framework in 2024. Nucleic Acids Res..

[59] Chen C., Wu Y., Li J., Wang X., Zeng Z., Xu J., Liu Y., Feng J., Chen H., He Y. (2023). TBtools-II: A “one for all, all for one” bioinformatics platform for biological big-data mining. Mol. Plant.

[60] Lescot M., Déhais P., Thijs G., Marchal K., Moreau Y., Van de Peer Y., Rouzé P., Rombauts S. (2002). PlantCARE, a database of plant cis-acting regulatory elements and a portal to tools for in silico analysis of promoter sequences. Nucleic Acids Res..

[61] Chao H., Li T., Luo C., Huang H., Ruan Y., Li X., Niu Y., Fan Y., Sun W., Zhang K. (2020). BrassicaEDB: A gene expression database for Brassica crops. Int. J. Mol. Sci..

[62] Jia Y., Yao M., He X., Xiong X., Guan M., Liu Z., Guan C., Qian L. (2022). Transcriptome and regional association analyses reveal the effects of oleosin genes on the accumulation of oil content in *Brassica napus*. Plants.

[63] Shannon P., Markiel A., Ozier O., Baliga N.S., Wang J.T., Ramage D., Amin N., Schwikowski B., Ideker T. (2003). Cytoscape: A software environment for integrated models of biomolecular interaction networks. Genome Res..

