# Supplementary material for: Genome-Wide Identification and Characterization of the UMAMIT Gene Family in Brassica napus: Evolution, Expression, and Potential Association with Oil Content
Source: Int J Mol Sci. 2026 Sep 26;27(19):8635. doi: 10.3390/ijms27198635 (PMC13665311; doi:10.3390/ijms27198635)
Supplement: Supplementary file 1 [file ijms-27-08635-s001.zip › Supplementary Files/Supplementary Files.pdf]

Supplementary Files

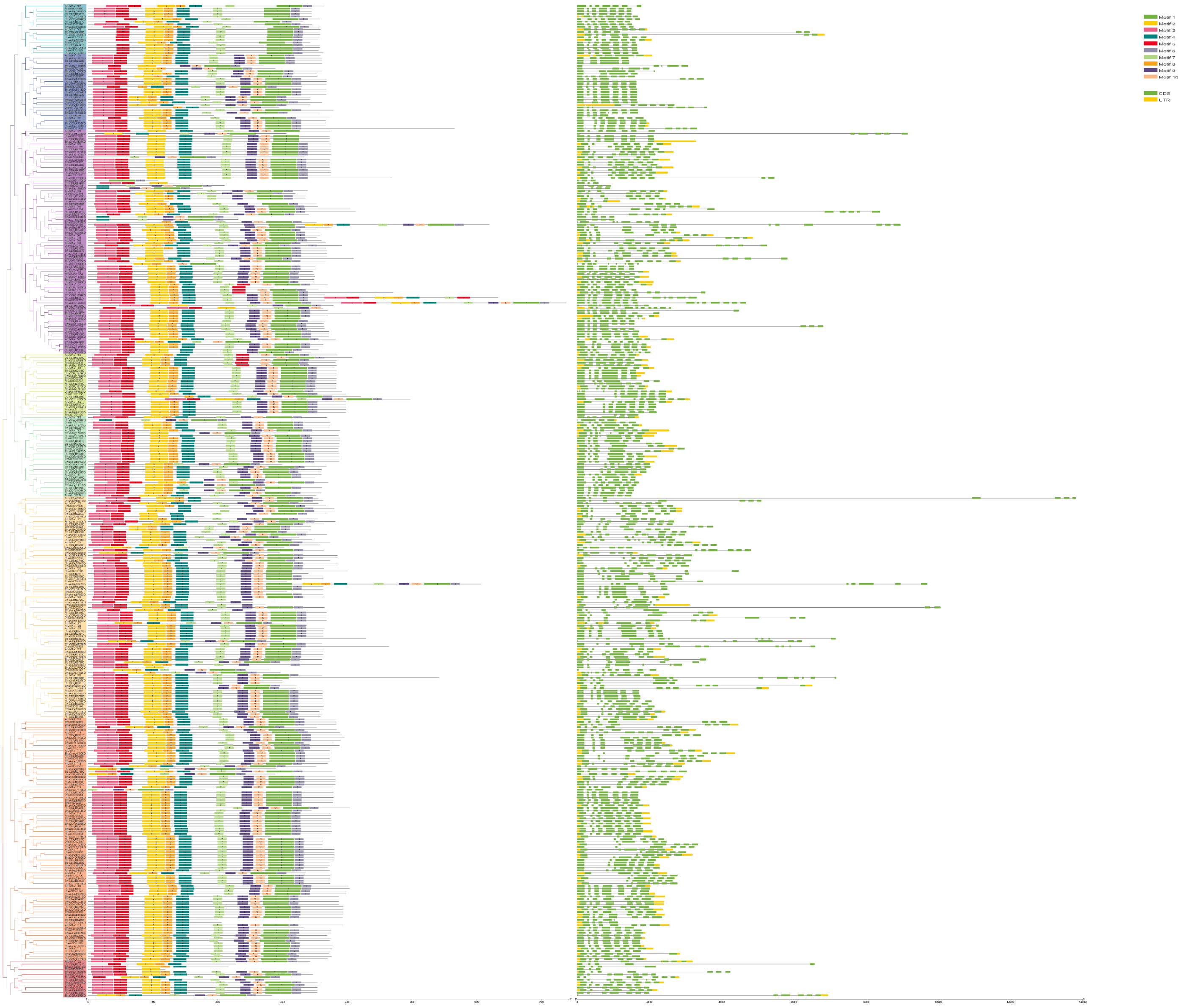

Figure S1 Phylogenetic tree (A), gene motifs (B) and gene structure (C) analysis of UMAMIT proteins from *A. thaliana* (44), *B. rapa* (78), *B. oleracea* (87) and *B. napus* (172). (B) Ten colors boxes represent ten motifs. (C) Green boxes, black lines, and yellow boxes indicate exons, introns, and untranslated regions, respectively.

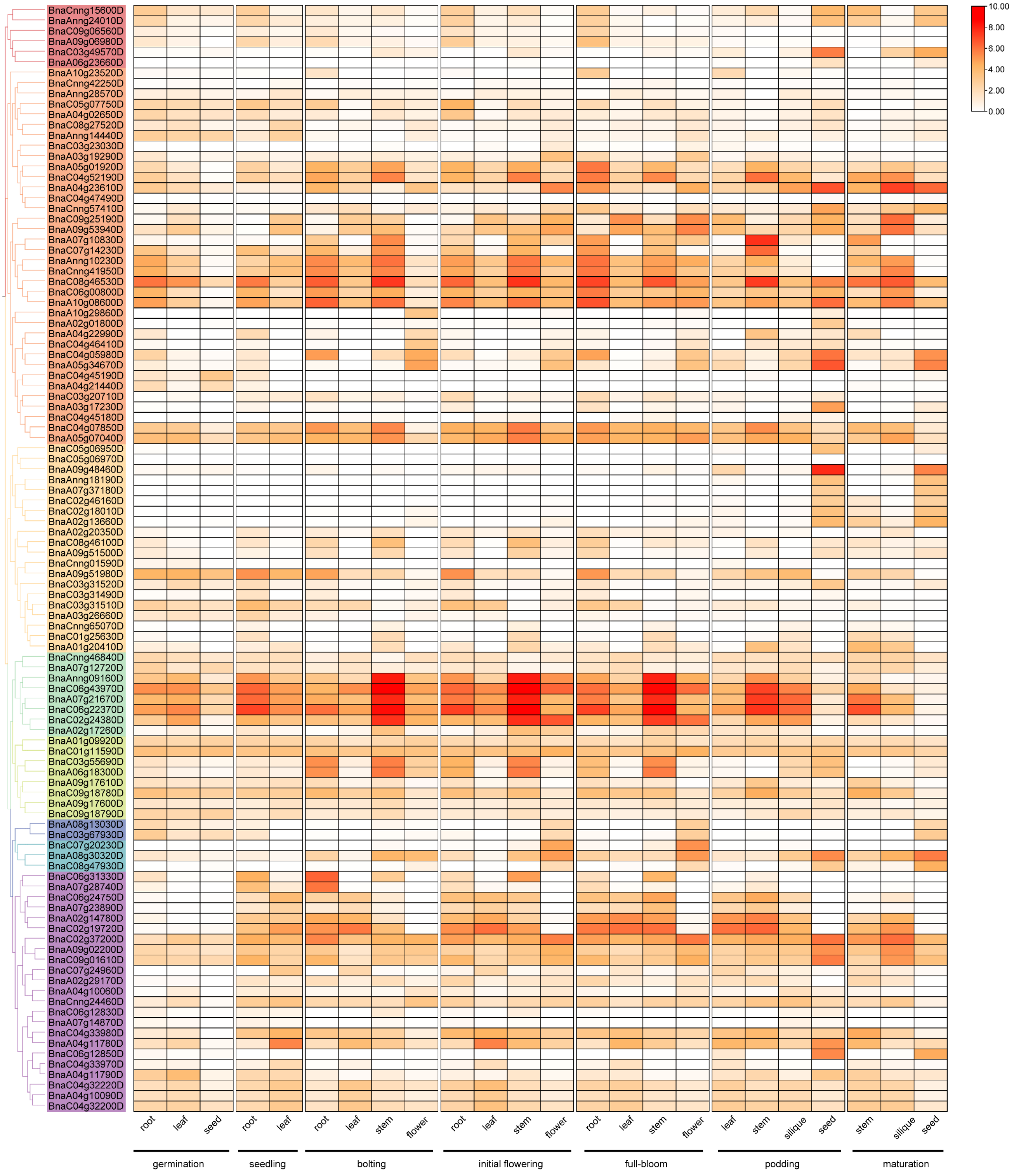

Figure S2 Expression patterns of *BnaUMAMIT* genes in different tissues at various developmental stages. The data comes from BrassicaEDB (https://brassica.biodb.org/ (accessed on 9 December 2023)). The heatmap is generated by comparing the relative results of different genes in different periods. Orange and white indicate high and low expression levels, respectively.

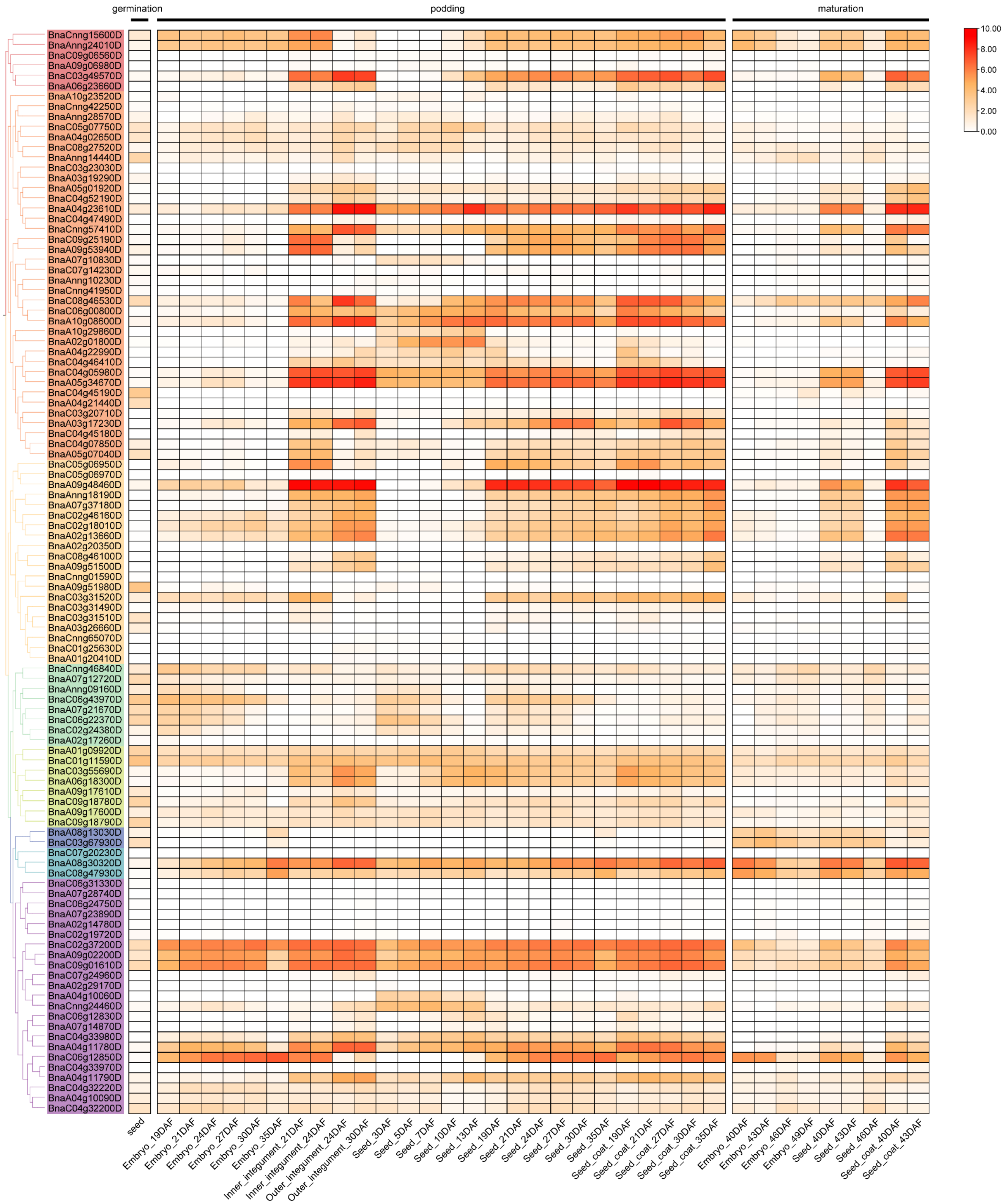

Figure S3 Expression patterns of *BnaUMAMIT* genes at different stages of seed development after pollination in *B. napus*.

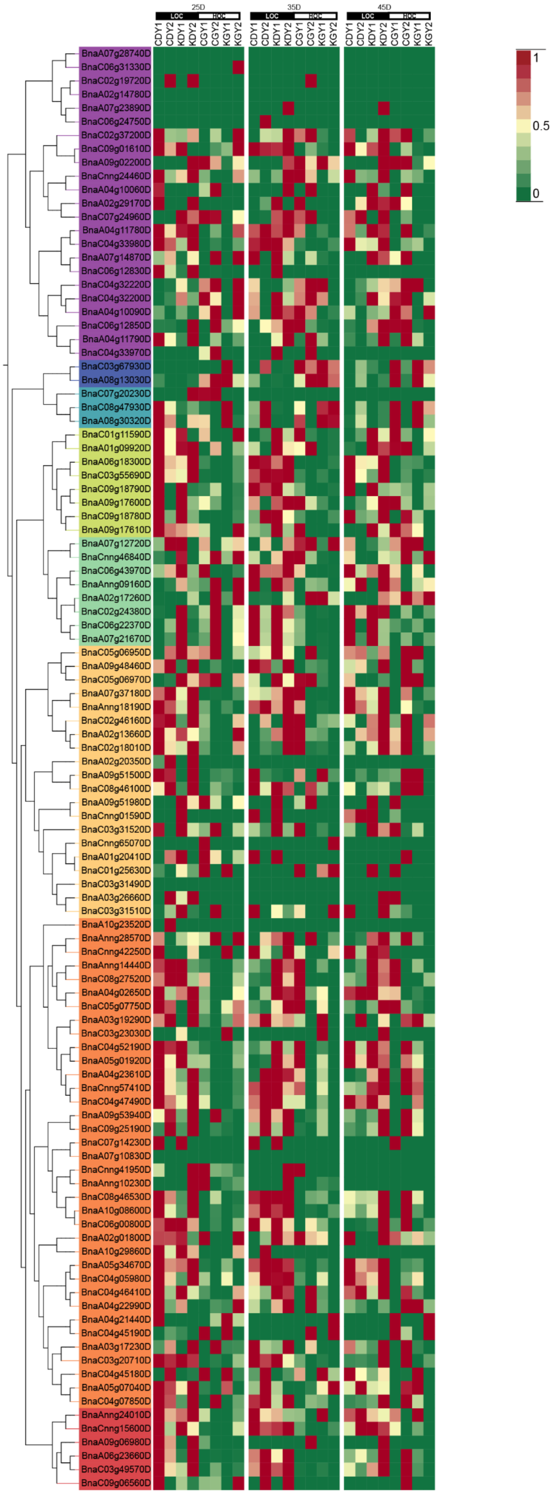

Figure S4 Expression patterns of *BnaUMAMIT* genes in high and low oil content *B. napus* seeds from 2 Regions at 25, 35, and 45 Days. LOC: low oil content, HOC: high oil content.CDY1/2 and KDY1/2 represent low oil content seeds; CGY1/2 and KGY1/2 represent high oil content seeds.

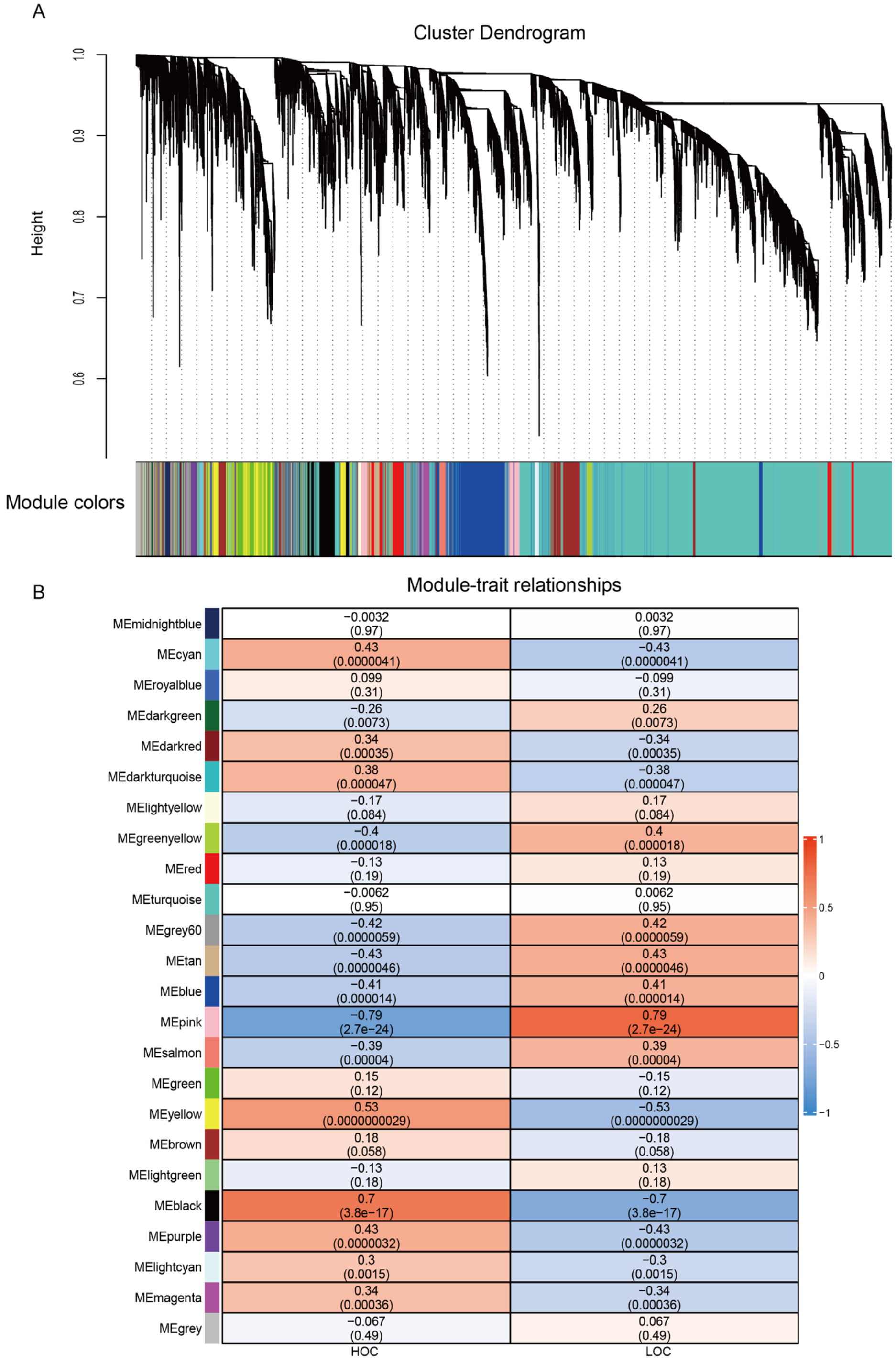

Figure S5 Weighted gene co-expression network analysis (WGCNA) in high and low oil content *B. napus* seeds. (A) Cluster dendrogram showing co-expression modules. Each leaf in the tree is one gene and modules corresponding to branches are labeled by different colors. (b) Heatmap of module-sample association depicting correlations between modules and the high low oil content. Each row corresponds to a module. The correlation coefficient and the corresponding *p*-value are shown in the matrix.

Table S1 Characteristics of *UMAMIT* genes in *A. thaliana*, *B. rapa*, *B. oleracea*, and *B. napus*.

Table S2 List of *UMAMIT* genes identified in *B. rapa*, *B. oleracea* and *B. napus*. The group have been assigned according to *Zhao et al*., 2021. The corresponding clade in *Zhao et al*., 2021 is indicated in parenthesis.

Table S3 Quantity distribution of *BnaUMAMIT* cis-acting elements.

Table S4 Primer sequences used in this study.
